# Holographic THz Beam Generation by Nonlinear Plasmonic Metasurface Emitters

**DOI:** 10.1021/acsphotonics.3c00775

**Published:** 2023-08-01

**Authors:** Symeon Sideris, Hu Zixian, Cormac McDonnell, Guixin Li, Tal Ellenbogen

**Affiliations:** † Department of Physical Electronics, School of Electrical Engineering, Tel-Aviv University, Tel Aviv 6997801, Israel; ‡ Center for Light-Matter Interaction, Tel-Aviv University, Tel-Aviv 6779801, Israel; § Department of Materials Science and Engineering, Southern University of Science and Technology, Shenzhen 518055, China; ∥ Institute for Applied Optics and Precision Engineering, Southern University of Science and Technology, Shenzhen 518055, China

**Keywords:** terahertz (THz), holography, metasurface, geometric phase, optical rectification

## Abstract

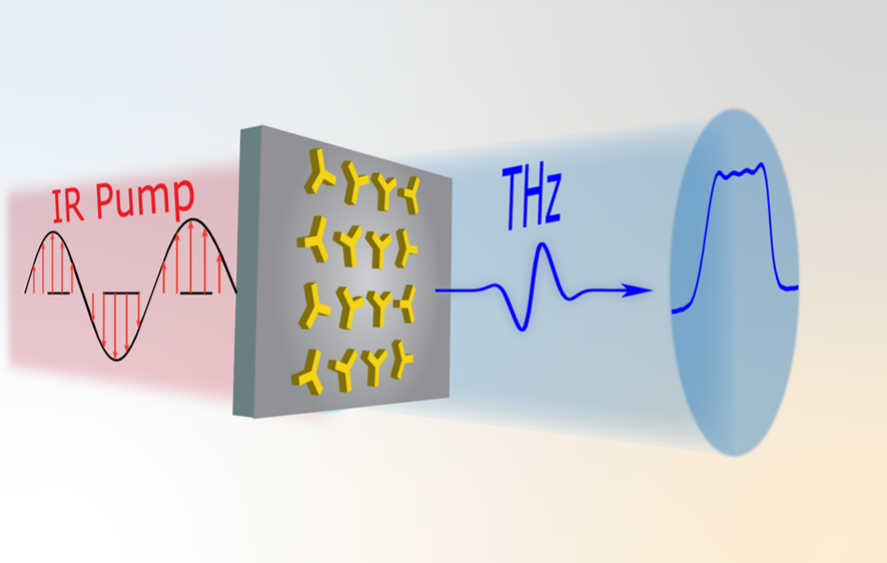

The advancement of terahertz (THz) technology hinges on the progress made in the development of efficient sources capable of generating and shaping the THz emission. However, the currently available THz sources provide limited control over the generated field. Here, we use near-field interactions in nonlinear Pancharatnam–Berry phase plasmonic metasurfaces to achieve deep subwavelength, precise, and continuous control over the local amplitude of the emitted field. We show that this new ability can be used for holographic THz beam generation. Specifically, we demonstrate the generation of precisely shaped Hermite–Gauss, Top–Hat, and triangular beams. We show that using this method, higher-order modes are completely suppressed, indicating optimal nonlinear diffraction efficiency. In addition, we demonstrate the application of the generated structured beams for obtaining enhanced imaging resolution and contrast. These demonstrations hold immense potential to address challenges associated with a broad range of new applications employing THz technology.

## Introduction

The spectral electromagnetic window in between the microwave and the far-infrared region, typically referred to as the terahertz (THz) frequency band, has attracted an ever-increasing interest over the past decades, inspiring the development of new multidisciplinary applications. Owing to the unique characteristics of many optically opaque materials in the THz regime originating from their molecular rotational and vibrational transitions, THz waves have been extensively applied in the advancement of nondestructive evaluation techniques.^1^ Exploiting these spectral fingerprints, THz radiation has been utilized in the sensing and identification of illicit substances,^2,3^ depth-resolved tomography,^4^ as well as security inspection.^5^ Additionally, due to the non-ionizing character of THz emission, recent studies have been devoted to noninvasive biomedical imaging^6,7^ and cancer cell identification.^8,9^ Moreover, envisioning the new generation of wireless communication systems, the adoption of THz frequencies is expected to play a pivotal role in enabling up to terabit data transmission rates.^10,11^

To expedite these technologies and realize the full potential of THz radiation, it is imperative to develop functional THz sources that offer controlled modal emission. In this regard, the adoption of structured light has fundamentally shaped recent progress in science and technology. For example, in the THz regime, structured illumination facilitates the emission of spatially tailored and nondiffracting beams, allowing for high resolution imaging, microscopy, and metrology schemes.^12–14^ In addition, electromagnetic waves carrying structured orbital angular momentum may promote the advancement of fast and secure optical communication systems.^15,16^ To effectively control and shape the THz emission, both passive and active configurations have been comprehensively studied over the years. Passive components such as diffractive optical elements provide various degrees of freedom on the post-generation shaping of the emitted wavefront.^17,18^ However, inherent material losses result in limitations to the operational bandwidth and difficulties in the integration to on-chip designs,^17^ introducing engineering constraints to the design of THz systems. On the other hand, dynamic control based on mechanical modulation is highly challenging, often requiring complex fabrication processes.^19^ Furthermore, the lack of efficient broadband electro-optical materials in the THz regime poses a fundamental challenge in the active shaping of THz emission.^20^ Currently, state-of-the-art initiatives involve the simultaneous generation and shaping of the THz emission by employing artificially nanostructured materials. For instance, the recently established spintronic emitters^21^ show promise in the ongoing endeavor of actively controlling THz radiation. To date, these emitters have exhibited binary phase control and polarization manipulations, enabling polarization switching, beam steering, and lensing.^22–25^ Another nanoscale approach on the generation of tailored THz emission is based on the utilization of nonlinear metasurfaces (NLMSs).^26–29^ More precisely, the irradiation of plasmonic metasurfaces with femtosecond laser pulses in close vicinity to their localized surface plasmon resonance induces strong single-cycle THz emission due to optical rectification.^26,30^ Interestingly, the recent expansion of the Pancharatnam–Berry phase (also called geometric phase) in the THz emission process from metasurfaces has aided in obtaining continuous spatial control over the emission phase.^31^ This was shown to demonstrate a variety of functional THz emitters.^32^ However, in order to obtain full control over the THz emission, both the phase and amplitude need to be locally controlled. In this work, we rely on near-field interference to obtain fine continuous local control over the THz amplitude and show that we can use this ability to demonstrate precise holographic THz beam generation using NLMSs.

## Design Principles

### Shaping the Near-Field THz Amplitude

**Figure 1:**
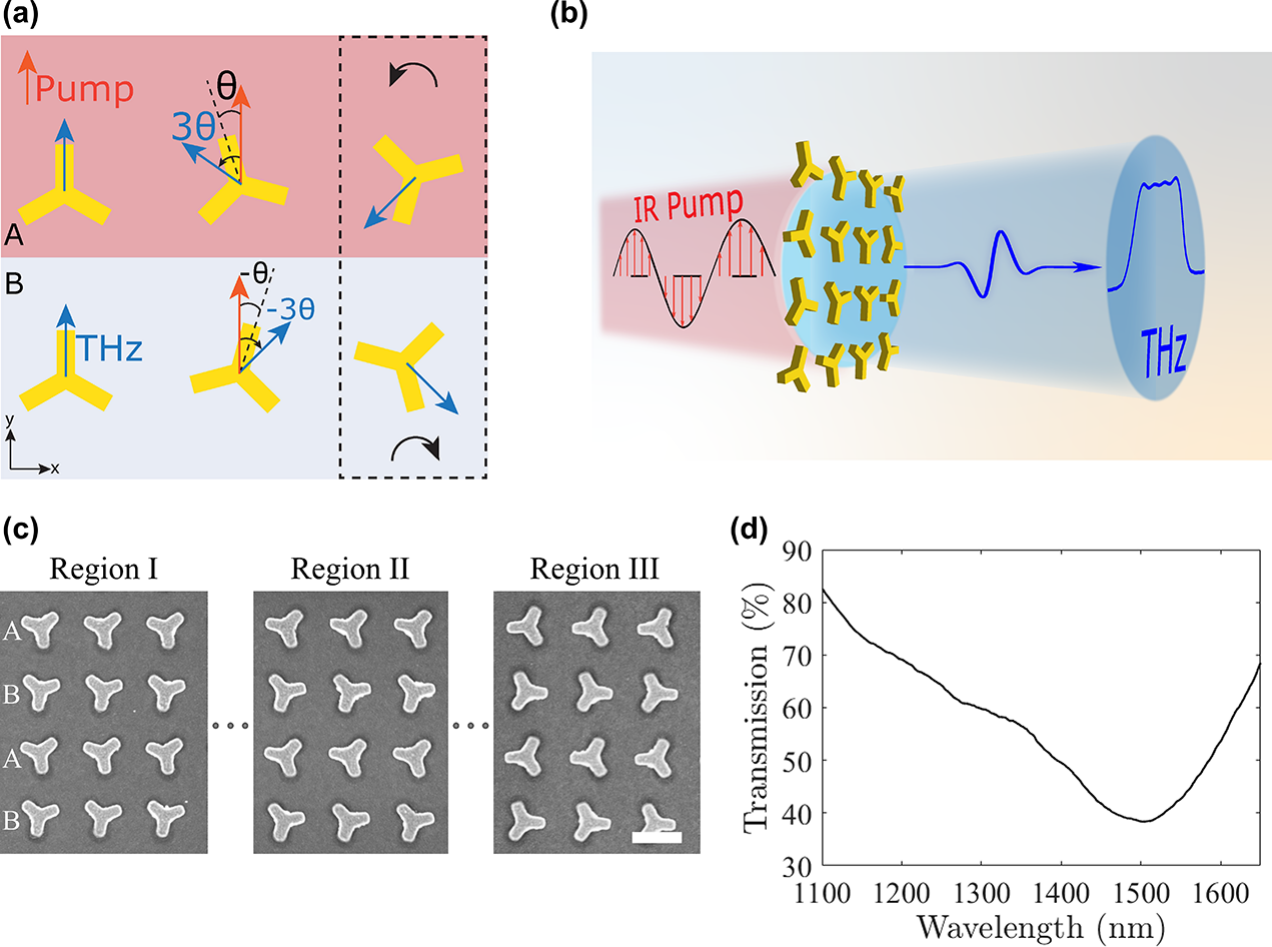
(a) Design principle of the controlled THz emission based on the near-field interference of adjacent *C*_3_ meta-atoms (dashed line). (b) Schematic illustration of the amplitude-controlled emission for the generation of 1D Fourier holographic images in the far-field. (c) Scanning electron microscopy imaging of 3 distinct regions contained in a holographic sample, where alternating rows A and B are continuously rotating. The scale bar is 500 nm. (d) Linear transmission measurement of a metasurface consisting of super-cells. The sample exhibits a broad resonance in the wavelength range 1200–1600 nm.

In order to attain rigorous control of the locally emitted THz amplitude, we devised a series of nonlinear plasmonic metasurfaces composed of *C*_3_ meta-atoms. The ensemble of meta-atoms consists of alternating rows A and B, where the nanoantennae are continuously rotated according to an arbitrary spatial function ± θ(*x*), respectively, where θ defines the angle between the principal axis of the *C*_3_ meta-atom and the *y*-axis (Figure 1a). Doing so, we establish a unit cell, referred to as “super-cell”, which consists of two adjacent meta-atoms as depicted in Figure 1a. The underlying mechanism of this design is based on the superposition of the near-fields emitted from each distinct unit cell contained in the super-cell. Specifically, assuming excitation with linearly polarized light at an angle ϕ with respect to the principal axis of the meta-atom, each *C*_3_ resonator emits linearly polarized THz waves along 3ϕ according to the selection rules that were previously reported.^31^ Thus, in the case of a linearly polarized pump along *ŷ* the emitted THz field from each *C*_3_ nanoinclusion is given by , where the sign of θ is alternating between the rows. The superimposed locally emitted THz field of the super-cell now reads *E*_THz_ = *ŷ* cos[3θ(*x*)], as the *x̂* polarized components of the field destructively interfere, while the *ŷ* components meet in phase due to the antiparallel rotation of the meta-atoms. Consequently, through meticulous selection of the spatial rotational function, the targeted complex spatial amplitude of the locally emitted THz wavepackets can be systematically tailored by
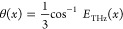
1

This technique is well suited for the precise holographic generation of THz beams, as illustrated in Figure 1b, where fine control over the local amplitude and phase is required. Notably, due to the extremely subwavelength scale of the meta-atoms relative to the wavelength of emitted THz waves, the modulated pixel size is 600 times smaller than the central wavelength of 1 THz, permitting the local shaping of light with unprecedented resolution.

### Far-Field Beam Shaping

Exploring the notion of such finely tailored emission, we designed a set of metasurfaces based on the principles of Fourier holography. Due to the extremely subwavelength scale of the meta-atoms relative to the emitted wavelength, the ensemble of super-cells constituting the metasurface can be treated as an electric field source, posing a spatially varying local field amplitude *E*_THz_(*x*), which is the kernel of the holographic image. Presuming the synchronous emission of THz waves from each super-cell, the spatiotemporal profile at the location of the metasurface (*z* = 0) is approximated as *E*_THz_(*x*,*t*,*z* = 0) = *E*_THz_(*x*)·*f*(*t*), where *f*(*t*) denotes the temporal shape of the emitted pulse. The decomposition of this near-field distribution to its corresponding spatial *k*_*x*_ and angular ω frequency components yields *E*_THz_(*k*_*x*_,ω,*z* = 0) = *Ẽ*_THz_(*k*_*x*_)·*F*(ω), where *Ẽ*_THz_ and *F* denote the Fourier transforms of the kernel and temporal shape of the pulse. During propagation along the *z*-axis, the wave accumulates a phase component of , which is mathematically described as
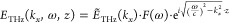
2

Collecting the light at a specific angle  in the far-field translates to the collection of specific momentum components . Thus, the radiated electric field amplitude in the Fraunhofer region is given by
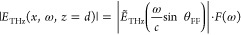
3indicating that the amplitude of the collected field exhibits a spectrospatial profile resembling the Fourier transform of the kernel function. Naturally, the lower frequency components are diffracted toward higher angles, resulting in the stretching of the shape in the spatial domain, as predicted by eq 3. Thus, rigorous manipulation of the near-field profile emitted by the metasurface enables the broadband generation of a wide range of beam shapes, as dictated by the mathematical principles of the Fourier transform.^33^

## Results and Discussion

### THz Generation and Detection

To experimentally demonstrate this concept, we fabricated and measured three Fourier holographic emitters. The fabrication steps are described in detail in Section S1 of the Supporting Information. As exhibited in the scanning electron microscopy imaging presented in Figure 1c, the samples consist of *C*_3_ meta-atoms, forming a square lattice with a constant period of 650 nm. The samples were fabricated using electron beam lithography on top of ITO-coated glass. According to preceding data, the nanofabrication of plasmonic metasurfaces on top of a thin ITO layer leads to an increase of the THz emission by 2–4 orders of magnitude relative to the case of a bare glass substrate owing to the epsilon-near-zero response of the ITO film.^28,30^ The samples exhibited a resonant response over the wavelength range of 1200–1600 nm, as shown in Figure 1d. In order to excite the localized surface plasmon resonance of the emitters, the samples were illuminated with linearly polarized light along a central wavelength of 1500 nm. The illumination was incident from the glass side of the metasurface, where ultrashort laser pulses of ∼50 fs pulse width activated the THz emission through optical rectification on the metasurface. The THz characterization was performed on a time-domain spectroscopy system (Figure S1), where the spatiotemporal overlap of the THz radiation with an infrared probe signal induced the electro-optical modulation of a ZnTe crystal, which offers a detection bandwidth of 2.5 THz. Due to the uniformity of the samples in the *y*-direction, the THz waveform was raster scanned solely along the *x* spatial position, enabling the extraction of the complex diffracted electric field profile in the far-field.

### Hermite–Gauss (1,0) Beam Generation

**Figure 2:**
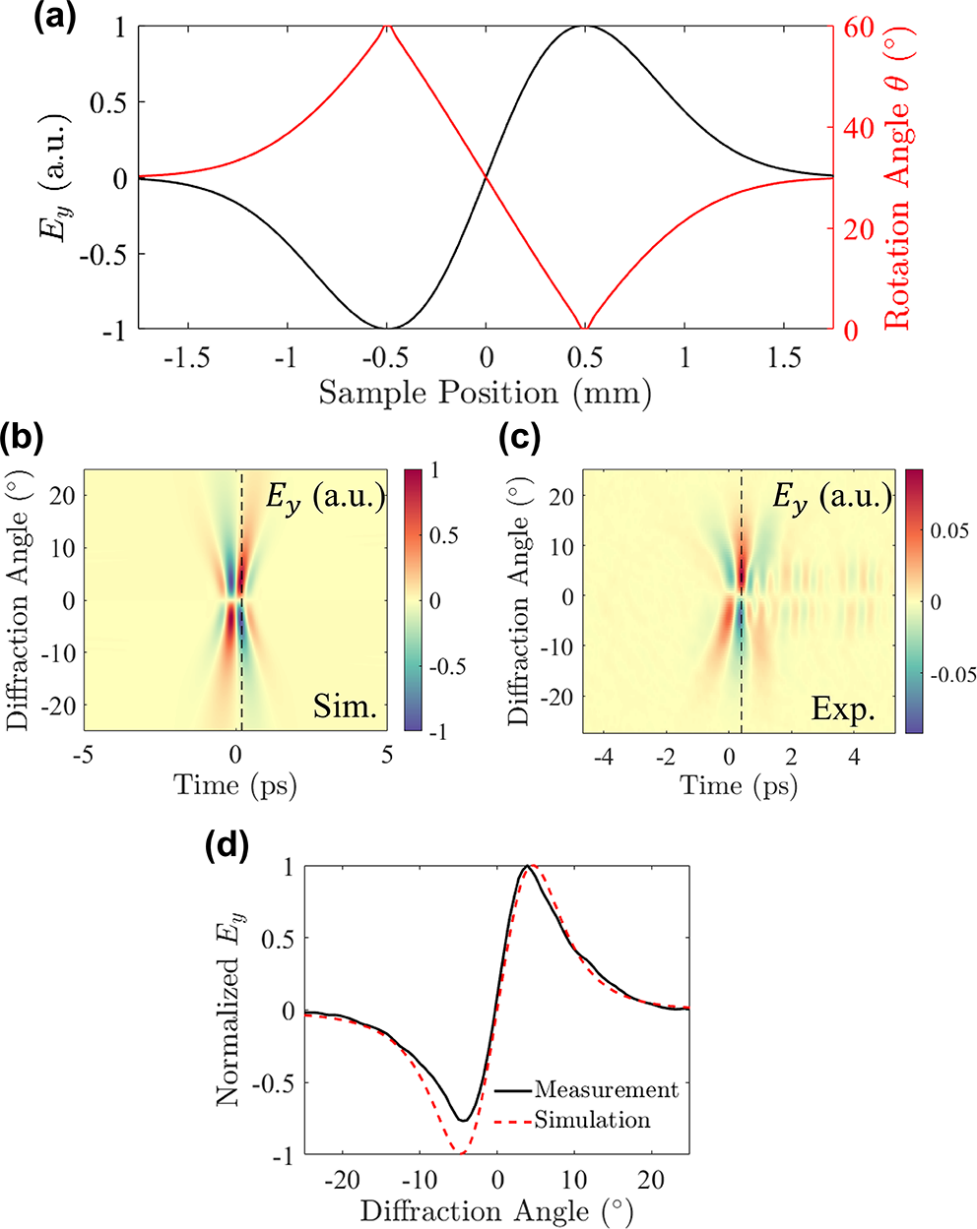
Spatiotemporal profile of a Hermite–Gauss (1,0) beam. (a) Design parameters toward the generation of a HG beam. The kernel function is mapped according to , where *a* = 0.7 mm. (b) Simulated and (c) experimental demonstration of the spatiotemporal profile of the emitted HG beam, exhibiting the emission of wavepackets that flip their phase transverse to the propagation. The dashed lines mark the (d) time-traces obtained at *t* = 0.2 ps, verifying the phase flip of the wavepacket. The experimentally obtained trace (black line) matched closely with the simulated (red dashed line) expectation.

The first holographic sample was designed to emit a Hermite–Gauss (1,0) (HG10) beam. To achieve this, the spatial orientations of the super-cells were carefully selected to map the complex near-field distribution according to , where *H*_1_ corresponds to the Hermite polynomial of first order. The design parameter *a* was chosen as 0.7 mm, and the sample covered an area of 3.5 mm × 1 mm in order to fit the emitted beam in our experimental numerical aperture (NA). The kernel function of the sample is presented in Figure 2a in addition to the corresponding spatial rotation function θ(*x*) required to match the local emission, calculated from eq 1. Using the beam propagation method, as described in eq 2, we performed simulations of the far-field THz emission from the metasurface. The simulated spatiotemporal response of the Hermite–Gauss beam is shown in Figure 2b, presenting the generation of a single cycle THz beam with the expected HG10 shape.

The fabricated metasurface was illuminated under a fluence of ∼210 μJ/cm^2^, and the temporal properties of the broadband field were measured by raster scanning of the wavefront in the collimated space (Figure 2c). Note that the spatial positions of the scanning slit and delay line have been translated into diffraction angles (*y*-axis) and time delay (*x*-axis), respectively. Here, the experimental spatiotemporal profile reveals the expected phase inversion of the wavepacket in the transverse direction relative to the propagation, exhibiting the excitation of two beam lobes with opposite phases. Sampling the wavefront in the image plane of our experimental apparatus, we observe the generation of a perfectly shaped HG along a constant time trace (Figure 2d), in very good agreement with the results obtained from the space to time mapping.

**Figure 3:**
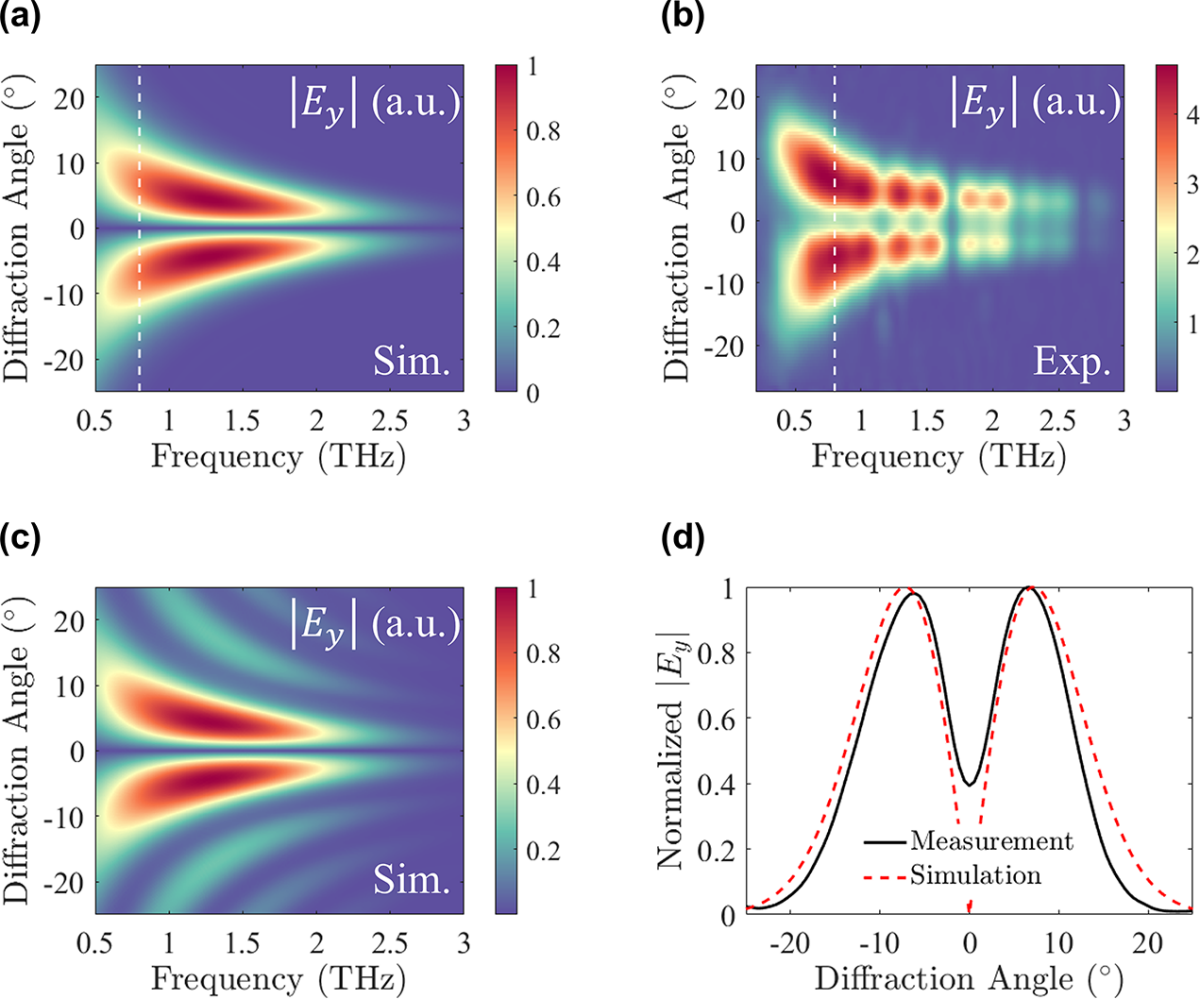
Spatiospectral profile of an HG (1,0) beam. (a) Simulated (normalized) and (b) experimental spectrospatial profiles obtained according to the Fourier transform of the spatiotemporal response of the sample. The profiles show broadband shaping of the THz field over the complete available bandwidth, expressed as the emission of two beam lobes. (c) Generation of a HG beam, following the illumination of a binary metagrating, where the meta-atoms flip their phase. The simulated sample consists of one period, and the sample’s size is set to 2.2 mm × 1 mm. (d) Comparison between the measurement (black) and simulation (red dashed line), demonstrating the emission of a perfectly shaped HG beam.

To further examine the response of the sample, we transformed the spatiotemporal response in the frequency domain. The extracted spatiospectral profiles are presented in Figure 3a,b, displaying the diffraction of the pulse into two beam lobes. It is important to note that the experimental beam profile in the spectral domain exhibits noticeable gaps, which are attributed to water vapor absorption consistently observed throughout our experimental findings.^34^ Moreover, it is interesting to highlight that previously generated HG beams were based on binary phase metagratings.^27^ However, the modal emission of such gratings was accompanied by the generation of higher-order HG modes, appearing as additional side lobes in the spatiospectral profile (Figure 3c). Here, such high-order modes are completely suppressed, indicating that the nonlinear diffraction efficiency into the desired mode reaches 100% due to the continuous and exact mode matching of the local THz field to the HG mode. Evidently, our approach results in the broadband shaping of the emitted light, covering all of the available bandwidth. Further assessing the quality of the generated beam, the spectral traces at 0.8 THz reveal an excellent agreement between the simulated and measured profiles, with the main difference lying on the nonzero crossing of the spectral response at the center of the sample (Figure 3d). This deviation from the simulated response originates from the finite size of the scanning slit in our experimental apparatus.

### Top-Hat Beam Generation

**Figure 4:**
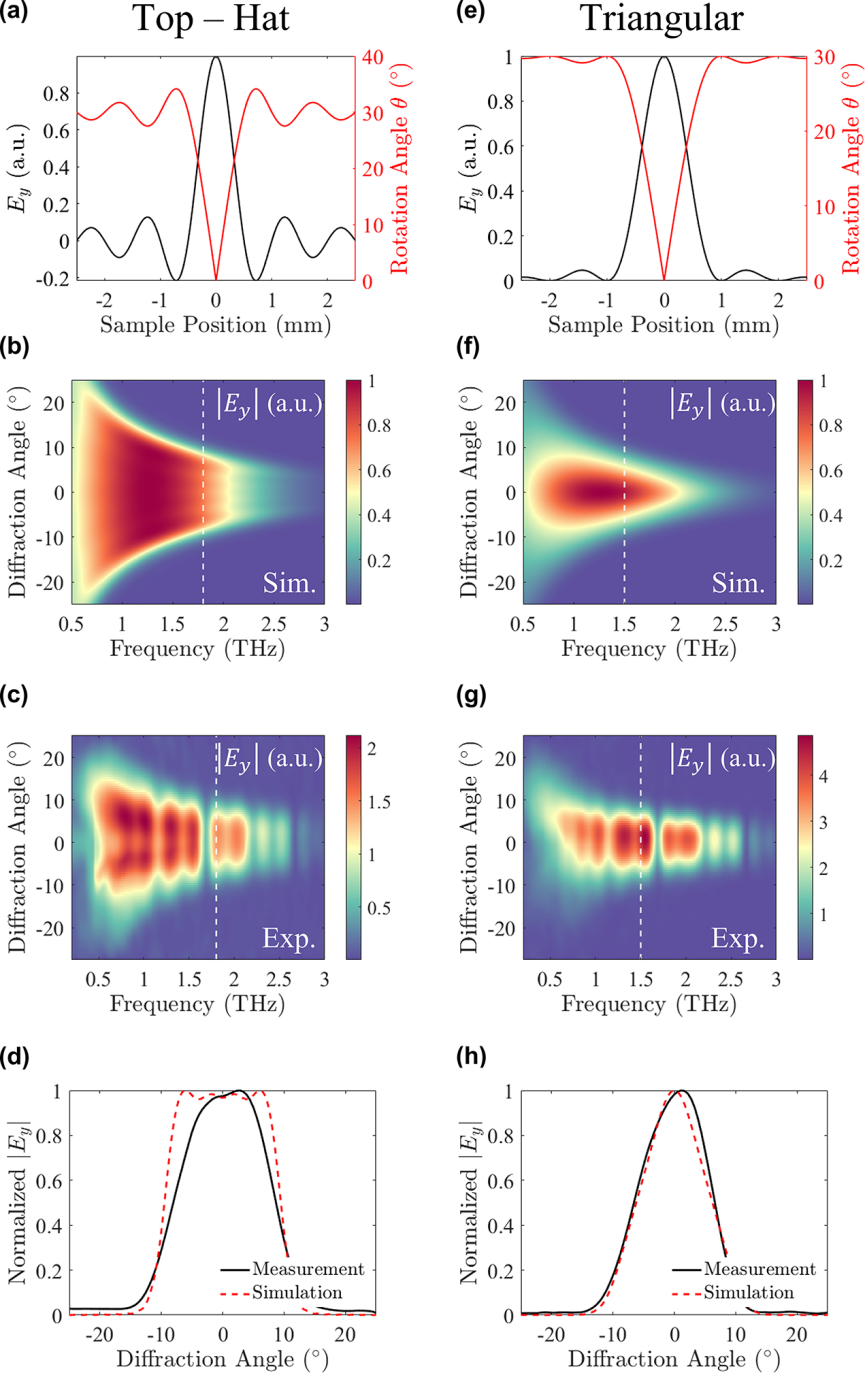
Generation of Top-Hat and Triangular beams. (a) Design function of a Top-Hat beam. The near-field intensity is mapped according to *E*_THz_ = sinc(*a*·*x*), with *a* = 2 mm^–1^. (b) The theoretical (normalized) and (c) experimental frequency components reveal the diffraction of a uniform intensity profile all over the available bandwidth. Examining the profile at 1.8 THz (white dashed line), we observe (d) good agreement between the simulation (red dashed line) and measured (black line) data, with an RMSE of 10%. (e) Design function of a triangular beam. The kernel is mapped according to *E*_THz_ = sinc^2^(*a*·*x*), where *a* = 1 mm^–1^. (f) Simulated (normalized) and (g) measured spatiospectral profiles of the triangularly shaped beam. To evaluate the quality of the emitted shape, a comparison is performed at 1.5 THz (white dashed line), (h) showing excellent agreement between the simulation (red dashed line) and measured (black) triangular profiles. A quantitative estimation based on the RMSE yields a 5% deviation between the simulated and measured profiles.

To show the ability of meticulously controlling the local THz amplitude for Fourier holography, we proceeded to the design of a Top-Hat beam, which corresponds to the radiation of a uniform intensity profile. In mathematical terms, a Top-Hat beam is represented in the spatial domain by a rectangular function , where *a* is the design parameter of the beam profile. To generate such a beam by Fourier holography, the holographic kernel is a sinc function, which requires intricate and continuous transitions from positive to negative local amplitude values. Once generated, such beams with a homogeneous intensity profile have numerous interesting applications. For example, they enable higher fields of view relative to conventional Gaussian profiles and thus may increase the imaging resolution, making them a highly desirable THz source.^35^ Previously, Top-Hat beams were generated using diffractive optical elements among others, which shape the beam to high-order super-Gaussian profiles.^36,37^ In the present study, the near-field amplitude was structurally matched to *E*_THz_ = sinc(*a*·*x*), where *a* = 2 mm^–1^, resulting in the conversion of the emission into a Top-Hat beam with a uniform intensity profile in the far-field. The design parameters of the sample are presented in Figure 4a, illustrating the required precision to generate the sinc kernel function. The emitter under study covered an area of 5 mm × 1 mm, and the emitted wavefront was measured in our experimental setup (Supporting Information Section S3). The spatiospectral profile depicted in Figure 4b reveals that the Top-Hat beam is diffracted over an extended spatial range. This outcome can be attributed to the higher spatial frequency components within its kernel, resulting in decreased energy density on the detector and thus lower detected signals. Detecting a beam profile of this nature poses a challenge given the high degree of accuracy necessary to measure a perfectly flat intensity profile. Initial evaluations of the Top-Hat beam reveal a deviation from the expected uniform profile. This deviation is credited to slight misalignment of the collection optics, affecting the balancing of the photodetector. In order to treat the experimental misalignment, we applied a post-processing step, which fixes the tilt in the frequency domain without affecting the diffraction limits of the beam (details in Section S5 of the Supporting Information). The experimentally acquired beam profile is presented in Figure 4c, in addition to a comparison performed at 1.8 THz (Figure 4d), showing the close resemblance between the simulated and experimental beam shapes.

### Triangular Beam Generation

Moving forward, we finally show that we can additionally design a triangularly shaped beam, which is the result of shaping the near field according to *E*_THz_ = sinc^2^(*a*·*x*). Here, the parameter *a* was selected as 1 mm^–1^, whereas the sample spans over an area of 5 mm × 1 mm. This configuration is intended to excite both the primary and secondary lobes of the modal intensity profile (Figure 4e). Despite the fact that the excited secondary lobes generate peaks of much lower intensity compared with the main lobe of the kernel function, their emission is related to the excitation of corrective terms that suppress higher-order modes. To examine the generated beam shape originating from the emission of a sinc^2^ kernel function, the spatiospectral maps of the simulated and experimental profiles are presented in Figure 4f,g, respectively. The profiles exhibit the generation of THz emission across a broad frequency range between 0.5 and 2.5 THz. The slight discrepancy between the theoretical and experimental profiles, manifesting in the asymmetric collection of the lowest frequency components, is due to minor misalignment in the collection optics and detection line. Nonetheless, the holographic beam quality is evaluated according to the root mean squared error (RMSE), indicating an ∼5–8% variance between the theoretical and experimentally obtained results in the spectral region of 0.8–2.3 THz (see Section S6 of the Supporting Information). Examining the cross section of the THz intensity at 1.5 THz (Figure 4h), we observe the generation of a perfectly shaped triangular holographic image, in excellent agreement with the simulated response.

### THz Imaging

**Figure 5:**
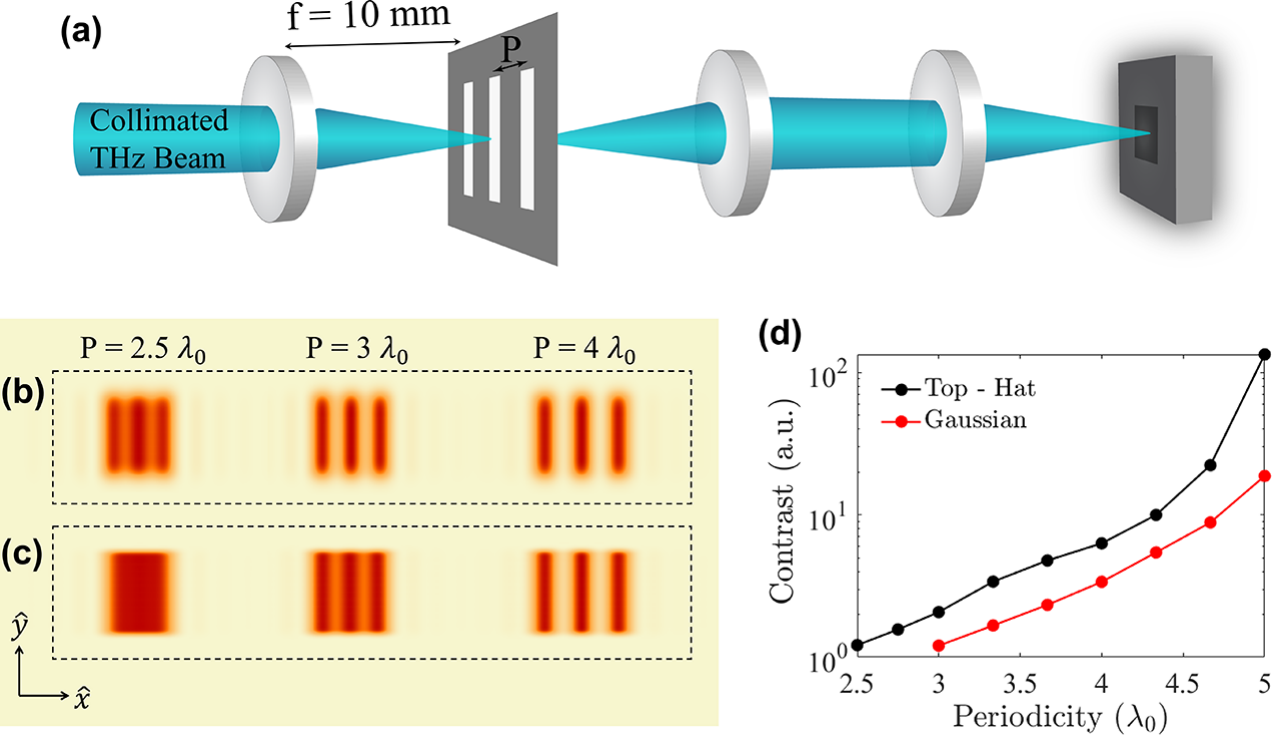
(a) Schematic representation of the simulated imaging system comprising a convergent lens (*f* = 10 mm), followed by a 4-f system forming the image on a detector scheme. The imaging targets consist of vertical slits of various periodicities (*P*), assuming a constant slit width (*w* = 300 μm). The simulated slits are infinite along *ŷ*. Reconstruction of the imaging targets for various periodicities *P*, assuming illumination with (b) Top-Hat structured illumination and (c) conventional Gaussian beam. (d) Contrast calculation benchmarking the imaging performance of the illuminating beams. Overall, the image reconstruction assuming illumination with a Top-Hat beam (black line) results in higher contrast values than a standard Gaussian beam (red line).

To illustrate the practical application of shaped emission, we conducted a simulation-based investigation. For this, we compared the imaging performance of the Top-Hat beam presented in Figure 4b and a standard Gaussian beam emitted from a 1 mm × 1 mm uniform metasurface as shown in our previous studies.^27^ Even though the NLMSs emit broadband THz beams, the imaging performance of the system presented in Figure 5a was evaluated for simplicity at the central frequency of 1 THz (λ_0_ = 300 μm). In this setup, both beams are focused using a converging lens (*f* = 10 mm), resulting in intensity profiles which correspond to a sinc and Gaussian profile along *x̂*, respectively, with similar half-width-at-half-maximum values at 1 THz. An imaging target is placed at the focal spot of the converging lens, followed by a 4-f system that collects the light diffracted within the NA of the system and focuses the captured signal on a detecting scheme. The image is reconstructed by raster scanning the sample on the *x*–*y* plane.

To benchmark the imaging performance of the two beam profiles under study, the sampling targets consist of 3 vertical slits, which are routinely used in THz imaging experiments.^12^ Here, the width of the slits remained constant (*w* = λ_0_), while the imaging performance was evaluated for various periodicities *P* between the slits. As presented in Figure 5b,c, sampling with a sinc function resulting from the Top-Hat beam yields enhanced imaging resolution along the axis of structured illumination, allowing distinction between imaging targets with periodicities as low as 2.5λ_0_. For the case of a Gaussian beam, small periodicities of 2.5λ_0_ result in a blurred image, where the slits are not distinguishable. Furthermore, the image reconstruction using the structured illumination of the target yields higher contrast values compared to the case of conventional illumination. To get a qualitative understanding of the contrast enhancement, we define the contrast as the ratio of maximal to minimal intensity values. Under this definition, the contrast values using the 1D Top-Hat illuminating source are estimated as 2.08 and 6.3 (a.u.) for the corresponding periodicities of 3λ_0_ and 4λ_0_ (Figure 5d). The respective values in the case of Gaussian illumination were calculated as 1.12 and 3.3 (a.u.), showcasing the decreased sharpness of the acquired images. Further investigation on the effects of the beam shape, as well as the adoption of more complex reconstruction algorithms may lead to additional enhancement in the lateral resolution and contrast of the images.

## Conclusions

In conclusion, we introduce a method to construct nonlinear plasmonic THz holographic emitters for precise amplitude beam shaping. To achieve this, we form super-cells consisting of two adjacent *C*_3_ meta-atoms, which encompass the notion of geometric phase. Exploiting the near-field interference of the meta-atoms contained in the super-cell, we are able to tailor the THz near-field with extremely high resolution, fitting 600 pixels in a single wavelength. This concept is applied to the design of Fourier holographic emitters, where the intricate and precise control of the local field results in perfectly shaped beam patterns with complete suppression of higher-order modes. We specifically demonstrated single-cycle, broadband, high-order Hermite–Gauss, Top-Hat, and triangular beams. Finally, a THz imaging system is simulated, which shows superior resolution and contrast when using structured light as opposed to conventional Gaussian beams. This work shows the basis for future development of complex metagrating emitters, where further rotation of the super-cells relative to the optical axis will allow simultaneous amplitude and phase control of the emission, introducing another degree of freedom in their design. We believe that this uniquely finely tailored control over the emission will allow the development of more compact and fully functional THz applications.

## Data Availability

The data that support the findings of this study are available from the corresponding author upon reasonable request.

## Supporting Information Available

Additional information on the sample fabrication, THz–TDS spectroscopy setup, spatiotemporal response, tilt-correction of the Top-Hat profile, and RMSE calculation (PDF)
