# Supplementary material for: Holographic THz Beam Generation by Nonlinear Plasmonic Metasurface Emitters
Source: ACS Photonics. 2023 Aug 1;10(8):2972–9. doi: 10.1021/acsphotonics.3c00775 (PMC10436349; doi:10.1021/acsphotonics.3c00775)
Supplement: Supplementary file 1 — ph3c00775_si_001.pdf [file ph3c00775_si_001.pdf]

Supporting Information

Holographic THz Beam Generation by Nonlinear Plasmonic Metasurface Emitters

Symeon Sideris,[1,2,*] Hu Zixian,[3] Cormac McDonnell,[1,2] Guixin Li,[3,4] and Tal Ellenbogen[1,2]

[1]*Department of Physical Electronics, School of Electrical Engineering, Tel-Aviv University, 6997801 Tel Aviv, Israel*

[2]*Center for Light-Matter Interaction, Tel-Aviv University, 6779801 Tel-Aviv, Israel*

[3]*Department of Materials Science and Engineering, Southern University of Science and Technology, Shenzhen, 518055, China*

[4]*Institute for Applied Optics and Precision Engineering, Southern University of Science and Technology, Shenzhen, 518055, China*

*symeons@mail.tau.ac.il

S1. Fabrication of the Metasurfaces

The metasurfaces used in this study were fabricated on top of the commercially available ITO-coated glass substrates. Initially, the substrates were cleaned using sonication when placed in acetone solution, isopropanol solution and deionized water, respectively. The substrates were then dried under a stream of nitrogen, followed by a baking process at 180 °C for 3 min. A thin film of a positive electron resist (2.45% PMMA, ALLRESIST) was spin-coated on top of the ITO-coated glass, which was then baked at 180 °C for 3 min. The patterns of metasurfaces were written using a standard electron beam lithography system. After developing the samples, a 30 nm thick gold layer was deposited on top of the photoresist using an electron beam evaporator. Finally, the plasmonic metasurfaces were formed after a lift-off process.

S2. THz–TDS Spectroscopy System

The THz response of the samples was extracted using the setup presented in Figure S1. The temporal profile was obtained using a motorized scanning stage, which controlled the time delay of the probe line. To extract the complete spatiotemporal profiles of the THz emitters at their Fourier space, a scanning slit of 3 mm width was placed in the collimated plane, following the collection of the THz emission by an off-axis parabolic mirror (f = 50.8 mm, Ø = 50 mm).

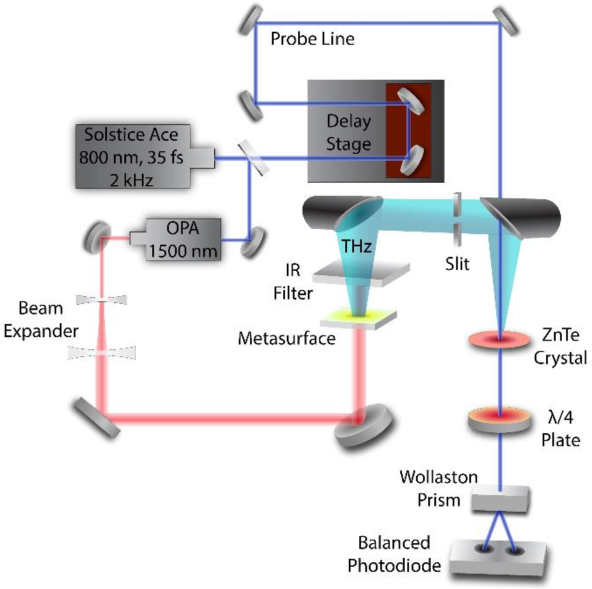

Figure S1. Illustration of time domain spectroscopy experimental setup. The metasurface is pumped by an optical parametric amplifier (OPA) emitting ultra-short pulses (~50 fs, 1500 nm) at a repetition rate of 2 kHz. The THz emission is collected and focused on a ZnTe crystal using a 4-f system comprised of 2 off-axis parabolic mirrors (f = 50.8 mm). The THz signal is electro-optically sampled in the ZnTe crystal through the spatiotemporal overlap with the probe line. The delay stage allows to measure the time domain shape of the THz pulse, while the scanning slit is used to extract the spatial profile of the beam.

S3. Spatiotemporal Response

S3.1 Top–Hat Beam

To generate the Top–Hat beam, the holographic function was designed as a sinc kernel function. As shown in Figure S2 (a) & (b), the detected signal is a single cycle pulse, which

maintains an almost flat intensity profile and abruptly goes to zero (Figure S2 (c)). Moreover, as seen in the simulated and measured spatiotemporal profiles, the top-hat pulse is diffracted in a higher spatial range due to the higher spectral components contained in its kernel function, relative to the previous samples.

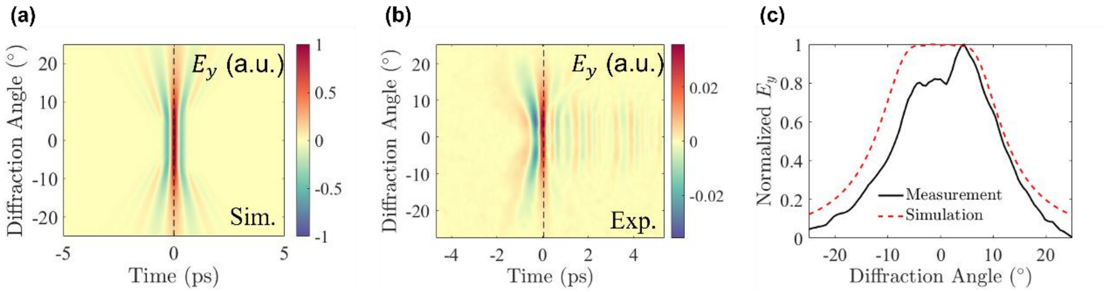

Figure S2. **Generation of a Top–Hat beam.** a) Theoretical and b) experimental spatiotemporal profiles following the emission of a sinc function to the far-field. The cross-section at the constant time trace of 0 ps reveals a c) flat electric spatiotemporal profile, where the simulated (red – dashed) response fits closely to the experimental (black) trace.

S3.2 Triangular Beam

The spatiotemporal response assuming the near-field mapping according to a sinc^2 kernel function is presented in Figure S3 (a) & (b). As we observe, this configuration leads to the generation of a single cycled pulse, with a pulse duration of ~1 ps, which corresponds to the central frequency of 1 THz, and is in line with previous experimental measurements.

Examining the spatiotemporal response of the sample, we observe that the wavepacket maintains a constant phase profile along the diffraction plane. To evaluate the quality of the emitted pulse, we initially compared the wavefront acquired experimentally with the result obtained from the space to time mapping. The comparison was performed at the constant time trace of 0 ps (dashed), disclosing a perfect agreement between the two, which manifest a triangular spatial intensity profile (Figure S3 (c)).

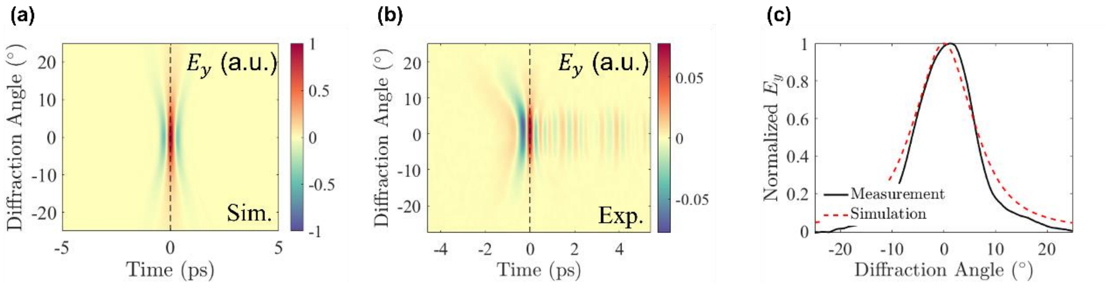

Figure S3. **Generation of Triangular beam.** Spatiotemporal profiles obtained through the a) space-time mapping and b) experimental procedure. The profiles demonstrate the emission of a single-cycle pulse where the wavepacket maintains a constant phase along the diffraction plane. The time-trace at 0 ps (black – dashed) displays a triangular intensity, which is identical in the experimental (black) and simulated (red – dashed) response.

S4. IR pump misalignment: Effect on measuring Top–Hat

In order to get an estimation resulting in the experimental error occurring at the measurement of the Top–Hat beam, we investigated the effect of misalignment of the pump beam on the

spectro-spatial profile. The intensity profile of the beam follows a Gaussian shape ($E_{\mathrm{pump}}(x) = e^{-2\frac{(x-x_c)^2}{w_0^2}}$), where x_c is the center of the beam and w_0 its beam waist.

Figure S4 shows that misplacing the beam relative to the center of the sample creates an asymmetric intensity profile. Yet, for small displacements (<2 mm) the generated asymmetry is relatively low, meaning that the origin of the error probably originates from the misalignment of the collection optics and the balancing of the photodetector.

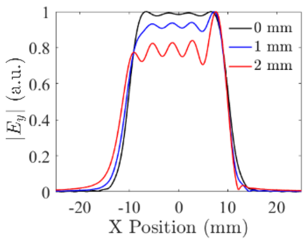

Figure S4. **Effect of displacing the pump beam relative to the sample's center.** As the displacement increases, the asymmetry observed in the spectro-spatial profile grows. The simulated frequency is 1.5 THz.

S5. Tilt correction on the Top–Hat spatiospectral profile

To correct the flatness of the measured Top-Hat beam, we applied the following algorithmic steps to each frequency component contained in the experimental dataset $E(x, f)$. Initially, we identify the spatial position of the intensity peaks (x_u and x_l) and define the line y_p connecting these points. This line's equation is of the form $y_p = \lambda x + b$, where $\lambda = (E_u - E_l)/(x_u - x_l)$.

To ensure the homogeneous intensity profile, we define the line y between (x_l, x_u) which satisfies: $y + y_p = E_u$. The final data-set containing the spectro-spatial intensity of the beam is given by $E_{\mathrm{fixed}}(x, f) = E(x, f) - \lambda(x - x_u)$, where $x \in [x_l, x_u]$, followed by a smoothing step.

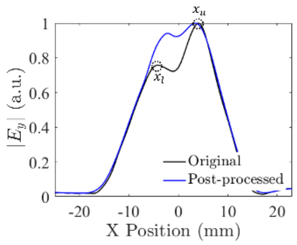

Figure S5. **Spectro-spatial tilt correction, applied to the Top–Hat beam.** The experimental data are shown in black, exhibiting the asymmetric detection of the emitted light. Following the application of the suggested algorithm between the points x_l and x_u, the post-processed profile has a more uniform intensity profile (blue) without affecting the data points outside of applied range.

S6. Beam shaping quality – RMSE calculation

An estimation of the quality of the emitted beam shape is given by the root mean square error (RMSE). The RMSE is calculated as a function of frequency (f) according to:

$$RMSE(f) = \sqrt{Avg[(E_{\mathrm{SIM}} - E_{\mathrm{EXP}})^2]} \qquad \text{(Eq. 1)}$$

where E_{SIM} and E_{EXP} denote the spatial intensity profiles of the beam shape obtained from the simulation and experimental data, accordingly. The operator $Avg[\ldots]$ indicates the spatial averaging of the function $(E_{\text{SIM}} - E_{\text{EXP}})^2$.

The RMSE evaluation of the Hermite–Gauss, Triangular and Top–Hat beams are presented below. In the case of the HG beam, we observe at Figure S6 (a) that the RMSE remains low (<10%) in the range of ~ 0.7 – 1.7 THz. For higher frequencies, the RMSE grows due to the asymmetry in the peak intensities of the generated lobes. Nonetheless, the generated beam maintains a low deviation relative to the expected profile. In Figure S6 (b), we present the RMSE calculated for the triangular shaped beam. Evidently, the generated beam shape corresponds to the expected value all over the available bandwidth. The RMSE is presented for the case of the Top – Hat beam (Figure S6 (c)). According to the calculated error estimation, the RMSE remains below 20% for the frequency components above 1 THz, which converges to a lower value for increasing frequencies. Surprisingly, there is a minor difference in the RMSE between the original and tilt corrected beam profiles. This suggests that the larger RMSE does not originate from the deviation from the uniform intensity profile, but rather from the sharpness in the transition between signal – no signal.

Finally, overall the RMSE exhibits its peak values for the lower frequency components due to the higher spatial spread of the generated beams, which adds to the complexity of the detection.

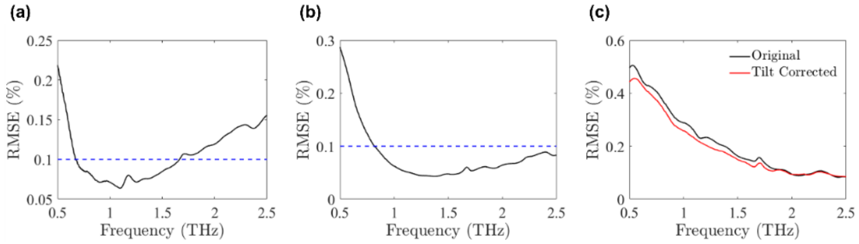

Figure S6**. Calculation of the RMSE for the generated beams.** a) Hermite–Gauss beam, b) Triangular beam and c) Top–Hat beam.
